# Cdk1 gates cell cycle-dependent tRNA synthesis by regulating RNA polymerase III activity

**DOI:** 10.1093/nar/gky846

**Published:** 2018-09-22

**Authors:** Maria C Herrera, Pierre Chymkowitch, Joseph M Robertson, Jens Eriksson, Stig Ove Bøe, Ingrun Alseth, Jorrit M Enserink

**Affiliations:** 1 Department of Molecular Cell Biology, Institute for Cancer Research, the Norwegian Radium Hospital, Montebello, N-0379 Oslo, Norway; 2 Centre for Cancer Cell Reprogramming, Institute of Clinical Medicine, Faculty of Medicine, University of Oslo, Oslo, Norway; 3 The Department of Biosciences, Faculty of Mathematics and Natural Sciences, University of Oslo, 0371, Norway; 4 Department of Medical Biochemistry, Oslo University Hospital, Oslo, Norway; 5 Department of Microbiology, Oslo University Hospital, Oslo, Norway

## Abstract

tRNA genes are transcribed by RNA polymerase III (RNAPIII). During recent years it has become clear that RNAPIII activity is strictly regulated by the cell in response to environmental cues and the homeostatic status of the cell. However, the molecular mechanisms that control RNAPIII activity to regulate the amplitude of *tDNA* transcription in normally cycling cells are not well understood. Here, we show that tRNA levels fluctuate during the cell cycle and reveal an underlying molecular mechanism. The cyclin Clb5 recruits the cyclin dependent kinase Cdk1 to tRNA genes to boost *tDNA* transcription during late S phase. At *tDNA* genes, Cdk1 promotes the recruitment of TFIIIC, stimulates the interaction between TFIIIB and TFIIIC, and increases the dynamics of RNA polymerase III *in vivo*. Furthermore, we identified Bdp1 as a putative Cdk1 substrate in this process. Preventing Bdp1 phosphorylation prevented cell cycle-dependent recruitment of TFIIIC and abolished the cell cycle-dependent increase in *tDNA* transcription. Our findings demonstrate that under optimal growth conditions Cdk1 gates tRNA synthesis in S phase by regulating the RNAPIII machinery, revealing a direct link between the cell cycle and RNAPIII activity.

## INTRODUCTION

The cyclin dependent kinase Cdk1 (also known as Cdc28) is the master regulator of the cell cycle in *Saccharomyces cerevisiae*. Cdk1 associates with nine different cyclins to execute the various stages of the cell cycle. A major function of Cdk1 is to activate cell cycle-dependent transcriptional programs by regulating the activity of various transcription factors (1). However, we and others have shown that Cdk1 also regulates the basal transcription machinery of RNA polymerase II (RNAP II) to stimulate the expression of many housekeeping genes upon cell cycle entry (2,3), which may be important for sustaining homeostasis as the cell forms a bud and expands its volume.

In addition to Cdk1, numerous other factors have been identified that affect cell cycle progression. For instance, depletion of Rpc17, as well as expression of mutant forms of Rpc53, both of which encode components of RNAPIII, lead to a significant G1 delay in yeast (4,5). Furthermore, in mammalian cells the expression of tRNAs has been shown to fluctuate during the cell cycle, and this is important for regulating the timely expression of certain cell cycle genes through codon usage preferences (6). However, whereas regulation of tRNA synthesis under conditions of cell stress has been relatively well studied (7), exactly how tRNA synthesis is regulated in unperturbed, cycling cells is less well understood.

The *S. cerevisiae* genome contains 275 tRNA genes (*tDNA*), which are transcribed by RNAPIII (8). tRNA genes contain an internal promoter that consists of a so-called A box and a B box, which are recognized by the basal transcription factor complex TFIIIC (8). TFIIIC is an approximately 500 kDa complex that consists of six proteins, including Tfc4. Binding of TFIIIC to the intragenic promoter allows for the assembly of TFIIIB from three subunits, TBP, Brf1 and Bdp1, which leads to recruitment of RNAPIII and activation of *tDNA* transcription (8,9). Genetic and biochemical studies have indicated that the Tfc4 subunit of TFIIIC is particularly important for recruitment of TFIIIB, making direct contact with Bdp1 and Brf1 (10,11). *In vitro* experiments have indicated that the main function of TFIIIC is to recruit TFIIIB, and that TFIIIB alone is sufficient for *tDNA* transcription (12,13). However, several studies have indicated that TFIIIC may contribute to reinitiation of RNAPIII on the same *tDNA* template to enhance transcriptional output (14–18). This is supported by early *in vitro* findings that TFIIIC is not released from the *tDNA* template during transcription (19). In fact, biochemical experiments in which TFIIIC was pre-incubated with one *tDNA* template, followed by addition of a second *tDNA* template and supplementing with the other essential components, only resulted in transcription of the first *tDNA* (19), demonstrating that TFIIIC retains RNAPIII on the *tDNA* template during transcription.

Because tRNA makes up 15% of the total cellular RNA pool, tRNA synthesis consumes a large portion of the cell's resources (20), and therefore RNAPIII activity is tightly regulated. A major regulator of RNAPIII is Maf1 (21), which is a transcriptional repressor that interferes with binding of RNAPIII to TFIIIB under unfavorable conditions (22–24). However, when conditions are optimal for cell growth, Maf1 is phosphorylated by several kinases, including TORC1, Sch9, PKA and CK2 (25). This leads to export of Maf1 from the nucleus and activation of RNAPIII (26). In parallel to Maf1, several cellular pathways directly regulate TFIIIB and RNAPIII activity, including the TORC1, PKA, CK2 and Sumo pathways (27–29). Furthermore, transcription of tRNA genes has been shown to fluctuate during the cell cycle, peaking in M phase (30), although the molecular mechanism underlying cell cycle-dependent *tDNA* transcription remains elusive.

Here, we studied cell cycle regulation of *tDNA* transcription and found that Cdk1 gates cell cycle-dependent *tDNA* transcription by enhancing the dynamics and activity of RNAPIII.

## MATERIALS AND METHODS

### Resources

#### Yeast strains and plasmids

*S. cerevisiae* strains were grown in appropriate media, depending on the experiment/genotype. Strains were derived directly from either the S288c strains RDKY3615 (31) or BY4741 using standard gene-replacement methods or intercrossing (see Supplementary Table S1 for strains and plasmids).

#### Antibodies

Anti-TAP antibody: RRID_AB_10709700, CAB1001, ChIP grade, rabbit polyclonal to TAP tag. Dilution 1:500; Anti-GFP antibody: RRID_AB_303395, ab290, ChIP grade, rabbit polyclonal to GFP tag. Dilution 1:500; Anti-c-Myc antibody: RRID_AB_627268, 9E10, mouse monoclonal to myc tag HRP conjugated. Dilution 1:1000; Anti-HA antibody: RRID_AB_307019, ab9110, ChIP grade, rabbit polyclonal to HA tag. Dilution 1:1000; Anti-Myc antibody: 9B11, ChIP grade, mouse magnetic bead conjugate. Dilution 1:20; Anti-HA antibody: 88836, ChIP grade, mouse monoclonal magnetic bead conjugate. Dilution 1:100. Protein molecular weight markers were used to verify the protein size.

### Experimental design and statistics

Information regarding sample size, error bars, and the number of biological replicates is given in the figure legends. *P* values were calculated using Student's *t*-test.

### Cell synchronization and flow cytometry

Cells were synchronized by culturing in YPD to log phase, after which 15 mg/l α factor was added for 3 h. Synchronized cells were released into the cell cycle by washing away the pheromone and resuspending the cells in YPD or in YPD + nocodazole (10 mg/l) to prevent initiation of a new cell cycle. For experiments that only required S phase-synchronized cells, cells were harvested after release from pheromone arrest when cells reached S phase based on microscopy. We observed day-to-day variation in the timing of cell cycle entry after release from pheromone arrest between different experiments, although there was little or no variation between cultures within each single experiment. Cell cycle arrest and synchronized cell cycle progression was confirmed by microscopy and by flow cytometry. Flow cytometry was performed as previously described (32).

### ChIP-Seq and ChIP-qPCR

The original Cdk1-TAP ChIP-seq dataset has been reported previously (2). ChIP-qPCR was performed as described previously (2,27). For primer sequences, see Supplementary Table S2. Visualization of read density was done in the integrative genome viewer (IGV) (33).

### RNA preparation and RT-qPCR

Total RNA purification and reverse transcription were performed as previously described (2,27). For primer sequences see Supplementary Table S2.

### Northern blot analysis

After heat denaturation at 55 °C for 5 min, total RNA samples were separated by 12% PAGE in 1× taurine at 200 V for 80 min. The tRNA was transferred to a nylon membrane (Hybond XL, GE Healthcare) by electroblotting in 1× taurine at RT for 90 min and 200 mA. RNA was UV-crosslinked to the membranes (120 mJ cm^−2^ in a CL-1000 UV-Crosslinker, UVP). Prehybridization, hybridization and washing steps were performed. ^32^P 5′-end-labeled oligonucleotides primers (Supplementary Table S2) complementary to the sequences of the initiator methionine, the non-intron region of tryptophan and leucine were used as probes. Hybridization signals were analyzed by phosphorimaging and ImageQuant TL software. Immature forms and other posttranscriptional modifications migrate slower than the mature forms. Hybridized probes were removed from the filters by boiling in 0.1% SDS. For primer sequences see Supplementary Table S2.

### Small-RNAseq

Small-RNAseq was performed as described previously (27) with the following modifications. Small RNA was purified from the RNA samples using the PureLink miRNA isolation kit (ThermoFisher Scientific), followed by sequencing using PE101 on HiSeq4000 (Illumina's HiSeq Xten, Hong Kong, China) including 10M clean reads per sample. Library preparation and sequencing was performed by BGI (Shenzhen, China). For RNA-seq data analysis, we used the *S. cerevisiae* genome sequence and associated annotation (R64-1-1.75) downloaded from Ensembl (35). We normalized the data by using ‘spike in’ standards (ERCC RNA spike in mix-4456740 Thermo Fisher for raw data normalization). Peaks were then annotated according to genomic location and the closest overlapping gene (36,37). We disregarded tRNAs encoded by mitochondria, because these tRNAs were not mapped in our original ChIPseq dataset (2), and therefore we could not draw conclusions on the effect of Cdk1 on expression of these *tDNAs*. Similarly, mature tRNA is decorated with modifications that may impair tRNA sequencing, and therefore our dataset is likely biased towards newly synthesized, unmodified tRNA.

### Mass spectrometry (MS)

MS experiments were performed as previously described (34) with minor modifications. Briefly, cells were synchronized as described above, and S phase cells were collected and fixed with 1% (vol/vol) formaldehyde for 30 min. Formaldehyde was quenched for 5 min by adding glycine to a final concentration of 125 mM. Cells were washed with cold Tris-buffered saline (50 mM Tris–HCl pH7.5, 150 mM NaCl) and lysed in lysis buffer [50 mM HEPES–KOH (pH 7.5), 100 mM KCl, 5 mM MgCl_2_, 5% glycerol, 1% Triton X-100, 3 mM DTT and protease inhibitor cocktail]. After centrifugation, supernatants were immunoprecipitated by mixing 800 μg of cross-linked material with 20 μl of magnetic beads covalently linked to the relevant antibodies. After overnight incubation at 4°C, beads were washed twice with high-salt TBS (50 mM Tris–HCl pH7.5, 350 mM NaCl) + protease inhibitor cocktail, twice with normal TBS + protease inhibitor cocktail and twice with normal TBS. MS samples were processed at the Proteomics Core Facility at Oslo University Hospital.

### Cell lysis, immunoprecipitation and Western blotting

Immunoprecipitation by western blotting was performed as previously described (2,27). For coimmunoprecipitations, S phase-synchronized cells were washed in ice-cold TBS, resuspended in lysis buffer [50 mM HEPES–KOH (pH 7.5), 100 mM KCl, 5 mM MgCl_2_, 1 mM EDTA, 1% Triton, 3 mM DTT, 5% glycerol, protease inhibitors, phosphatase inhibitors] and lysed by vortexing with glass beads followed by centrifugation to remove cell debris. Equal amounts of proteins were used for immunoprecipitation using magnetic beads conjugated covalently to relevant antibodies. After extensive washing with lysis buffer, coimmunoprecipitated proteins were resolved by SDS-PAGE and analyzed by western blotting with the indicated antibodies.

### Phos-tag gel analyses

Proteins from synchronized cells samples, prepared as previously described, were precipitated by using TCA. After washing twice with 100% ethanol, proteins were resuspended in 1 M Tris–HCl pH 8 buffer. Equal amounts of proteins were used to load the gels. After heat denaturation at 95 °C for 5 min, debris was discarded by centrifugation and loaded into the gel. Ten percent acrylamide Phos-tag gel electrophoresis run for 6 h at 150 V (Supersep, Wako chemicals), followed by three washes for 10 min with transfer buffer containing 5 mM EDTA and three washes for 10 min with transfer buffer without containing EDTA. The gel was transfered to a membrane and phosphorylation of Bdp1 was finally monitored by western blotting as described previously (38) using Myc antibodies.

### Fluorescence loss in photobleaching (FLIP) assay and data analysis

5–10 μl of synchronized and released cells were added to a 0.5–1 mm thick minimal media agar slab on a microscope slide, covered with a 0.17 mm objective glass, sealed with VALAP around the edges, and placed in a pre-heated cage type microscope incubator at 30 C. Imaging and bleaching was performed on an inverted Leica DMI6000 microscope with a TCS SP8 scanner equipped with a white light laser, using an 100× 1.4 NA Plan-Apo objective. The following scan settings were used; zoom: 10×, pinhole: 2 AU, pixel size: 70 nm, optical section 896 nm, line averaging: 3, scan speed: 1800 Hz, pixel dwell time: 3× 825 ns. Excitation of GFP was done at 490 nm and 2% laser intensity. Bleaching was done with 482 nm light at 100% laser intensity (measured to ≈100 μW). The GFP emission was collected from 495 to 569 nm with a time-gated HyD-detector in the time window of 0.3–12 ns after excitation.

FLIP assay data acquisition was performed in a single-blinded manner, the person responsible for imaging was not aware of which strain was being analyzed. Image acquisition procedure: (i) Find cell in correct cell cycle phase based on morphology. (ii) Define a rectangular bleach region of interest (ROI) covering 50% of the nucleus. (iii) Start imaging and image for 10 frames. (iv) Activate 482 nm laser at 100% in the bleach region. (v) Image at 2 Hz for 200 frames, with bleaching active in bleach ROI.

Image analysis pipeline (macro file available on request). All image analysis was performed with ImageJ (v. 1.51.n) in the following manner: (a) A polygonal ROI was manually fitted around the nucleus (Nucleus ROI). (b) A fluorescence loss (FL) ROI was created by subtracting the bleached region from the Nucleus ROI (FL ROI). (c) A copy of the FL ROI was placed outside of the cell for local background fluorescence measurement (Background ROI). (d) The mean intensity and the standard deviation in each ROI for each time point in the time lapse was measured.

Data analysis pipeline (R code available on request): All fluorescence intensity data was imported in to R (v 3.4.1), reshaped with dplyr (v. 0.7.2), and plotted with ggplot2 (v. 2.2.1). Fluorescence intensity was background subtracted and normalized to the average intensity at the first frame of bleaching. Curve smoothing was performed by calling the ‘geom_smooth()’ function of ggplot2 with default arguments, which defaulted to a smoothing method of ‘*gam*’ (Generalized Additive Models).

### *In vitro tDNA* pull-down experiments

To immobilize the *tDNA* template, biotinylated primers were used to amplify the *tL(CAA)C* gene from the plasmid pBS-SK-*tRNA^LEU3^* (39). For primer sequences see Supplementary Table S2. 10 μg of biotinylated *tDNA* was incubated with 1 mg Dynabeads M-280 Streptavidin (Invitrogen) in TEN buffer (5 mM Tris–Cl (pH 7.5), 0.5 mM EDTA, and 1 M NaCl) at 24°C for 24 h with mixing at 1400 rpm. Beads were washed for three times with TEN buffer and blocked by incubating with 5 mg/ml BSA in TEN buffer for 1 h at 4°C while rotating. A PCR fragment amplified from a plasmid containing the *BRAF1* gene (pLVX-*BRAF1*) was used as a negative control. Next, S phase-synchronized cells were lysed in lysis buffer [50 mM HEPES–KOH (pH 7.5), 100 mM KCl, 5 mM MgCl_2_, 1 mM EDTA, 5% glycerol, 1% Triton X-100, 3 mM DTT and protease inhibitor cocktail]. Extracts were centrifuged at 13000 rpm for 15 min at 4°C to remove debris, after which total protein concentration was measured using the Bradford protein assay. The volumes of the extracts were adjusted to attain equal volumes and protein concentrations. Finally, 2 μg of immobilized *tDNA* was incubated with 0.5 mg protein extract for 20 min at 24°C while mixing at 950 rpm. The beads were then washed three times with 300 μl of either lysis buffer containing 100 mM KCl, in lysis buffer lacking KCl but containing either 200 mM NaCl or 350 mM NaCl, and pulldowns were analyzed by SDS-PAGE followed by Western blotting with the indicated antibodies.

## RESULTS

### Cdk1 is recruited to *tDNA* genes to promote cell cycle-dependent *tDNA* transcription

Using ChIP-seq we have previously shown that Cdk1 localizes to at least 200 genes to promote their transcription during cell cycle progression (2). While most of these genes were protein-coding genes, we noticed that Cdk1 was also present at at least 20 dubious ORFs, such as *YHR145C, YJL007C* and *YOL013W-A* (2). Interestingly, careful inspection of the data revealed that most of these ORFs were located in close proximity to tRNA genes (*tDNA*) or snoRNAs (Supp. Dataset 1). For instance, *YHR145C, YJL007C* and *YOL013W-A* overlap *tM(CAU)J2, tP(UGG)H* and *tP(UGG)01*, respectively. These small non-coding genes were omitted from the original analyses due to filtering by the bioinformatics software. We therefore manually inspected this ChIP-seq dataset and discovered that Cdk1, in addition to localizing to protein-coding genes, is also present at nearly all RNAPIII-transcribed genes, i.e. most of the *tDNAs* (Supplementary Figure S1A) as well as *RDN5-1, RPR1, SCR1, SNR6* and *SNR52*, (Supplementary Figure S1B). We validated these ChIP-seq results by ChIP-qPCR using primers that either recognize all anticodons of initiator methionine *tDNA* (*tDNA^iM^*) or the introns of all immature tryptophan *tDNA* (*tDNA^W^*) anticodons (Supplementary Figure S1C). *PMA1, SSE1* and an untranscribed region of *ChrV* were used as positive and negative controls (Supplementary Figure S1C), because we have previously shown that these genomic regions recruit either high levels of Cdk1 (*PMA1*) or little or no Cdk1 (*SSE1*), whereas the region of *ChrV* recruits no detectable Cdk1 at all (2). Together, these data show that Cdk1 is significantly enriched at RNAPIII-transcribed genes, including *tDNA*.

**Figure 1.**
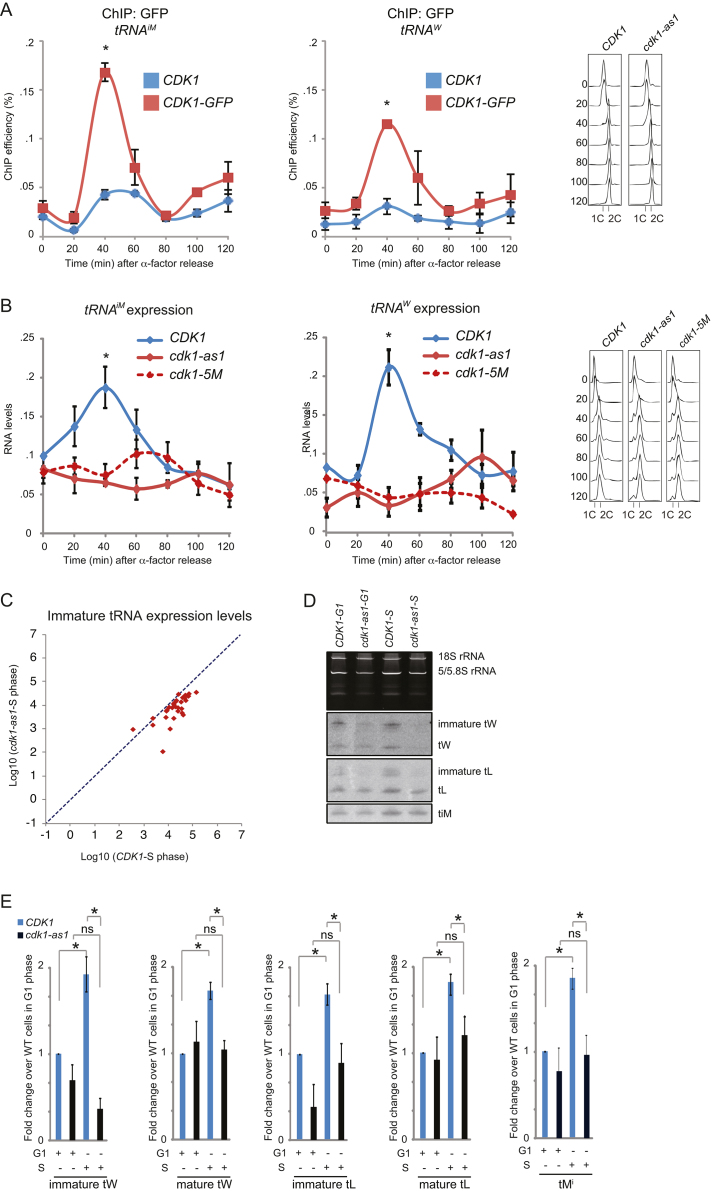
Cdk1 is recruited to RNAPIII-transcribed genes during S phase to boost *tDNA* transcription. (**A**) Cdk1 levels at tRNA genes peak in S phase. Untagged (*CDK1*) and GFP-tagged (*CDK1-GFP*) strains were synchronized in G1 phase using pheromone, washed and released into YPD. Samples were taken at the indicated time points and localization of Cdk1 to *tDNA^iM^* and *tDNA^W^* was monitored by qPCR. Values are given as percentage of input. Error bars indicate SEM of three independent experiments. Asterisk, *P* < 0.05. Cell cycle analysis by flow cytometry is indicated in the panel to the right. (**B**) Analysis of *tRNA^iM^* and immature *tRNA^W^* expression levels during the cell cycle. WT *(CDK1), cdk1-as1* and *cdk1–5M* strains were synchronized and sampled as in (A) and RNA levels were analyzed by RT-qPCR. For each strain values were normalized to time-point zero after alpha factor release. Error bars indicate SEM of three independent experiments. All *P* values were calculated using Student's *t*-test. (**C**) Scatter plot of the small RNA-seq dataset showing log10 FPKM tRNA values in *cdk1-as1* mutants versus WT cells for intronic (immature) tRNAs only. RNA was isolated from S phase cells (confirmed by flow cytometry) and analyzed by small-RNA-seq. The y-axis scale represents log_10_ of the FPKM in *cdk1-as1* strain and x-axis represents log10 of the FPKM in *WT* strain, both in S phase. Data was normalized by using spike-in RNA standards. Dashed line indicates slope = 1, and the majority of tRNAs fall below this line, indicating reduced tRNA expression in *cdk1-as1* mutants compared to WT cells. (**D**) Northern blot analysis of tRNA levels. Total RNA was isolated from synchronized cells (either G1 or S phase) of the indicated genotype. Primers that recognize the sequences of *tRNA^iM^* and non-intronic regions of *tDNA^W^* and *tDNA^L^* were used as probes. The upper graph shows an ethidium bromide gel with 18S, 5.8S and 5S rRNA. (**E**) Quantifications of the Northern blot shown in (D). Error bars indicate SEM of three independent experiments. Asterisk, *P* < 0.05; ns, not significant.

Because Cdk1 is the master regulator of the cell cycle, we hypothesized that it localizes to tRNA genes to promote their transcription during the cell cycle. To test this hypothesis, we first performed a cell cycle arrest-release experiment in which we followed Cdk1 recruitment to tRNA genes in synchronized cells. Interestingly, we observed a sharp increase in Cdk1 levels at tRNA genes ∼40 min after release from α factor-induced G1 arrest (Figure 1A, see Dataset 2 for data for all qPCR experiments). Cell cycle analysis of these samples by flow cytometry revealed that Cdk1 recruitment was highest during late S phase (Figure 1A); note that late S phase overlaps with early M phase events in *S .cerevisiae* (40). Interestingly, recruitment of Cdk1 to these tRNA genes coincided with increased *tDNA* transcription (Figure 1B). These findings were specific, because (i) neither Cdk1 recruitment to *SSE1* nor *SSE1* expression levels changed significantly in these same samples during the time course (Supplementary Figure S2A and B) and (ii) ChIP-qPCR using a control strain expressing Rub1-GFP (*S. cerevisiae* Nedd8), which is a ubiquitin family member that is rapidly proteolytically processed at the C-terminus (41) to release free GFP, which served as a negative control, did not show elevated recruitment to tRNA genes during S phase (Supplementary Figure S2C).

The fact that Cdk1 recruitment to tRNA genes correlates with increased tRNA expression levels suggests that Cdk1 gates tRNA synthesis during S phase. We tested this hypothesis by analyzing *tDNA* transcription during the cell cycle using two different *cdk1* alleles: *cdk1-5M* and *cdk1-as1. cdk1-5M* encodes a temperature-sensitive form of Cdk1 with severely reduced kinase activity already at permissive temperatures (42). The *cdk1-as1* allele encodes a form of Cdk1 in which the gatekeeper phenylalanine at position 88 is replaced with a much smaller glycine residue. This creates an enlarged ATP binding pocket that can accommodate the bulky, non-hydrolysable ATP analog 1-NM-PP1, which inhibits the kinase activity of Cdk1 (43). However, the gene product of *cdk1-as1* has a ten-fold reduction in ATP-binding affinity and a 6-fold reduction in maximum ATP turnover rate (43), resulting in a profound hypomorphic phenotype even in absence of 1-NM-PP1 (e.g. see (32,44)).

Using these two alleles we studied *tDNA* transcription through the cell cycle in synchronized cells. Because Cdk1 activity is important for cell cycle entry, the experiment was performed at permissive temperature (*cdk1-5M*) and in absence of 1-NM-PP1 (*cdk1-as1*), so that there was sufficient Cdk1 activity for cells to initiate the cell cycle (32). Interestingly, even though Cdk1 activity is only partially reduced in these two mutants, the brief peak in *tDNA* transcription observed in wild-type (WT) cells during late S phase occurred neither in *cdk1-5M* mutants nor in *cdk1-as1* mutant cells (Figure 1B). These data show that full Cdk1 activity is required for cell cycle-dependent tRNA expression.

To determine whether Cdk1 only regulates a specific subset of *tDNA*s, or whether Cdk1 has a more global impact on tRNA synthesis, we studied tRNA levels in WT cells and *cdk1-as1* mutants during S phase by RNAseq of small RNAs only (small-RNAseq, see Materials and Methods). Reads were normalized using spike-in of RNA standards to allow for direct comparison between the two strains. We focused on expression levels of the 61 yeast tRNAs that contain introns, because these are the immature, newly transcribed forms. There were in total 54 intronic tRNAs present in our small-RNAseq dataset (Supp. Dataset 3, *TAB1*). Strikingly, 47 out of these 54 immature tRNAs (87%) were expressed at lower levels in *cdk-as1* mutants, and the median expression level of all 54 intronic tRNAs was reduced by nearly 50% (log_2_ = −0.977) in *cdk1-as1* mutants relative to WT cells (Figure 1C and Supp. Dataset 3, *TAB1*). These results are consistent with our RT-qPCR data showing that Cdk1 activity promotes tRNA expression (Figure 1B). We found that expression of non-intronic tRNAs was also decreased in *cdk1-as1* mutants (Supp. Dataset 3, *TAB2*), which was also apparent from our RT-qPCR experiment with the intronless tRNA^iM^ (Figure 1B, left panel). This might be surprising, because tRNAs are thought to be very stable. However, studies in bacteria have indicated that under certain conditions tRNAs can be highly unstable (45); furthermore, it has been shown that in yeast a very large fraction of newly synthesized tRNA is rapidly degraded soon after synthesis (46). One explanation for our findings is therefore that the cell briefly induces a spike in tDNA transcription, followed by degradation of a large amount of the immature tRNA. In addition, it should be noted that analysis of tRNA levels by RNAseq and RT-qPCR may be biased towards unmodified and newly synthesized tRNA, because modifications that occur during tRNA maturation can pose roadblocks that affect the efficiency of tRNA sequencing (47). Nonetheless, these data indicate that Cdk1 broadly affects *tDNA* transcription in S phase, consistent with our finding that Cdk1 physically localizes to nearly all *tDNA* genes (Supplementary Figure S1A).

We validated our RT-qPCR and small-RNAseq data by Northern blotting using primers that recognize the total pool of *tRNA^iM^, tRNA^W^* and *tRNA^L^*, respectively, including mature and immature forms. While in WT cells the pool of *tRNA^iM^*, as well as immature *tRNA^W^* and *tRNA^L^*, were significantly increased in S phase compared with G1 phase, *cdk1-as1* mutant cells completely failed to increase tRNA levels in S phase (Figure 1D and E), confirming our RT-qPCR results shown in Figure 1B. These data show that Cdk1 activity is required for the cell cycle-dependent increase in *tDNA* transcription, thereby contributing to homeostasis of the tRNA pool.

We also aimed to understand whether transcriptional regulation by Cdk1 through the cell cycle includes other class III genes, as suggested by the ChIP-seq data (Supplementary Figure S1B). We isolated total RNA from WT cells and *cdk1-as1* mutants in G1 and S phase, and analyzed *SNR52* and *SCR1* expression by RT-qPCR analysis. Indeed, we found that transcription of these two RNAPIII-transcribed genes was also cell cycle dependent and regulated by Cdk1 (Supplementary Figure S2D and E). Together, these data show that Cdk1 regulates RNAPIII activity at RNAPIII genes, although for the remainder of this study we will focus on tRNA genes.

### Clb5 recruits Cdk1 to tRNA genes

**Figure 2.**
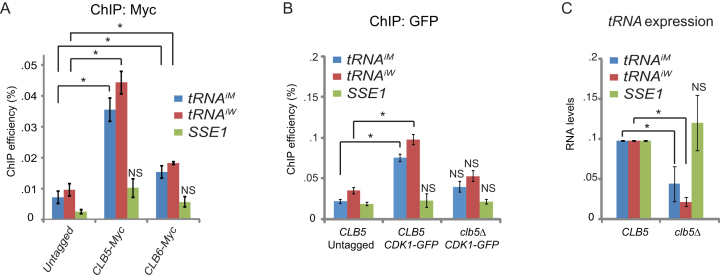
The cyclin Clb5 recruits Cdk1 to tRNA genes to stimulate *tDNA* transcription. (**A**) Analysis of cyclin recruitment by ChIP-qPCR. Untagged cells or cells expressing *CLB5-MYC* or *CLB6-MYC* were grown to log phase and analyzed by ChIP-qPCR using anti-Myc antibodies and primer pairs for *tRNA^iM^, tRNA^W^*, and *SSE1* (negative control (2)). Values are given as percentage of input. Error bars indicate SEM of three independent experiments. Asterisk, *P* < 0.05. NS, not significant. (**B**) *CLB5* is required for recruitment of Cdk1 to tRNA genes. Untagged WT cells, WT cells expressing *CDK1-GFP*, and *CDK1-GFP*-expressing *clb5Δ* mutants were grown to log phase and analyzed by ChIP-qPCR using anti-GFP antibodies and primer pairs for *tRNA^iM^, tRNA^W^*, and *SSE1*. Values are given as percentage of input. Error bars indicate SEM of three independent experiments. Asterisk, *P* < 0.05. NS, not significant. (**C**) *CLB5* is required for efficient tRNA synthesis. WT and *clb5Δ* strains were grown to log phase and *tRNA^iM^* and immature *tRNA^W^* levels were determined by RT-qPCR. Values were normalized to WT. Error bars indicate SEM of three independent experiments. Asterisk, *P* < 0.05. NS, not significant. All *P* values were calculated using Student's *t*-test.

Cdk1 associates with different cyclins to execute its various cell cycle-dependent functions. To identify which cyclin mediates the cell cycle-dependent recruitment of Cdk1 to *tDNA* we systematically analyzed recruitment of cyclins to *tRNA^iM^* and *tRNA^W^* by ChIP-qPCR (Figure 2A and Supplementary Figure S2F). Mirroring our finding that transcription of *tDNA* peaks during late S phase, we observed that the S phase cyclin Clb5 was significantly enriched at *tDNA* (Figure 2A). Clb6, the other S phase cyclin, was also significantly enriched at tRNA genes, although its levels were much lower than those of Clb5. In contrast, the non-S phase cyclins Clb1, Clb2, Clb3, Clb4 and Cln1 were undetectable at *tRNA^iM^* and *tRNA^W^* genes (Supplementary Figure S2F). These data suggest that Clb5 is the main cyclin important for the S phase-dependent increase in *tDNA* transcription. Indeed, Cdk1 was no longer recruited to *tDNA* in mutants lacking *CLB5* (Figure 2B). This resulted in a significant reduction in the expression of *tRNA^iM^* and *tRNA^W^*, whereas expression of the control gene *SSE2* was unaffected (Figure 2C). These data show that Clb5 mediates the recruitment of Cdk1 to *tDNA* to induce S phase-dependent *tDNA* transcription.

### Cdk1 regulates *tDNA* transcription independently of Maf1

**Figure 3.**
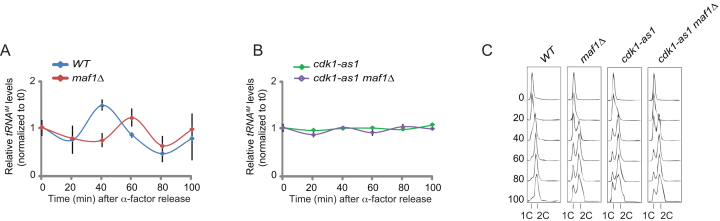
Deletion of *MAF1* does not restore cell cycle-dependent *tDNA* transcription in *cdk1-as1* mutants. (**A**, **B**) *tRNA^iM^* expression levels were analyzed in WT cells and in *maf1* mutants (A), and in *cdk1-as1* and *cdk1-as1 maf1* mutants (B) as described in Figure 1B. Values were normalized to WT time-point zero after alpha factor release. Error bars indicate SEM of three independent experiments. (**C**) Cell cycle analysis of the same samples shown in (A) and (B) by flow cytometry.

A major regulator of *tDNA* transcription is the transcriptional repressor Maf1. Under conditions favorable for cell growth, several kinases phosphorylate and inhibit Maf1 to promote *tDNA* transcription. Therefore, we investigated the possibility that Cdk1 stimulates cell cycle-dependent *tDNA* transcription through inhibition of Maf1. We performed a time course experiment where we monitored *tDNA^iM^* transcription in synchronized cells. Both WT cells and *maf1Δ* mutants showed increased *tDNA* transcription after release from G1 phase (Figure 3A). The peak in transcription appeared to be delayed in *maf1Δ* mutants, which, for reasons that are presently unclear, was caused by delayed release from G1 phase (Figure 3C). *cdk1-as1* mutants failed to induce the spike in *tDNA* transcription during S phase (Figure 3B), but more importantly, deletion of *MAF1* did not restore the peak in *tDNA* transcription in the *cdk1-as1* mutant background. These data suggest that Cdk1 promotes cell cycle-dependent *tDNA* transcription independently of Maf1.

### Cdk1 activity increases Tfc4 levels at tRNA genes

**Figure 4.**
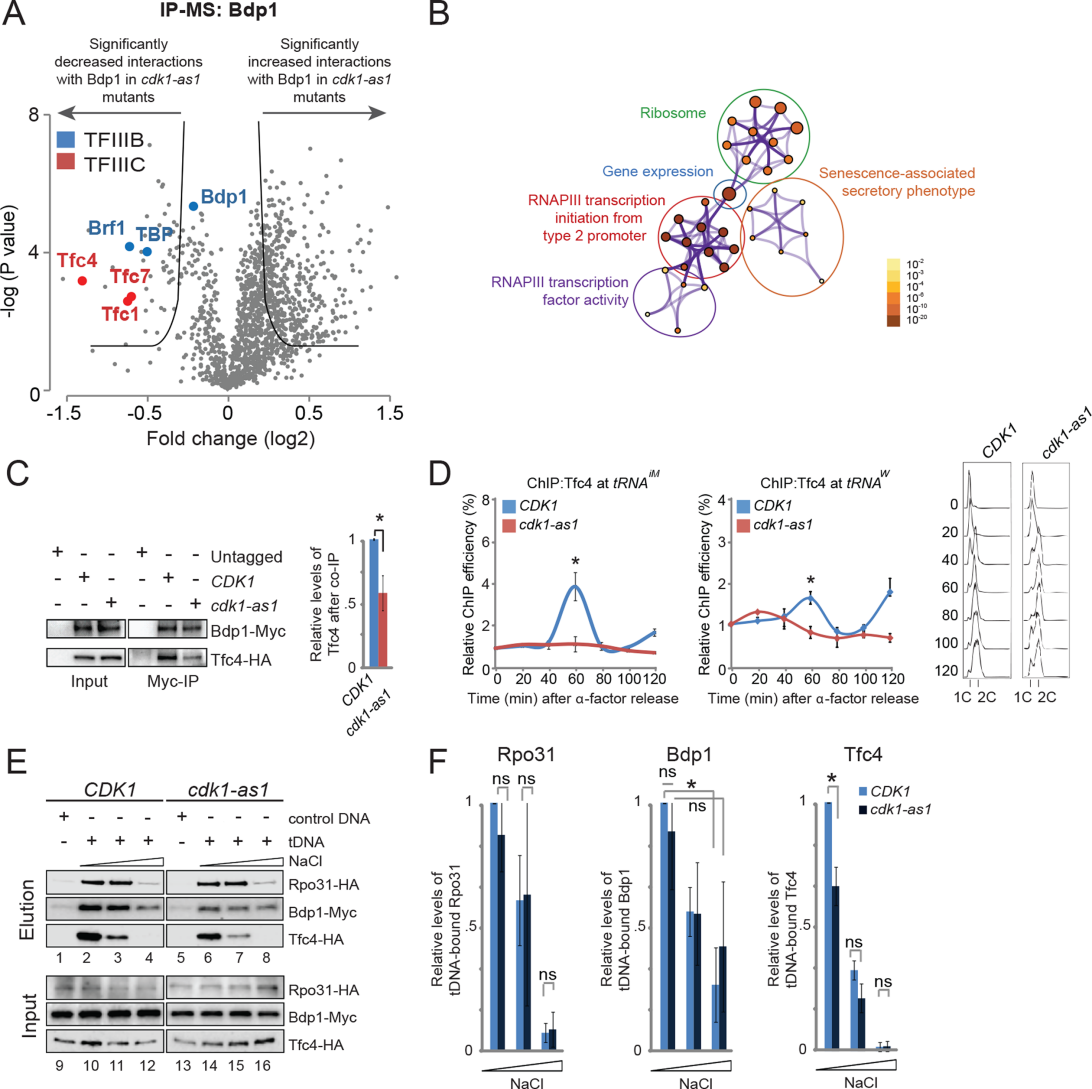
Cdk1 promotes the interaction between Bdp1 and Tfc4. (**A**) Volcano plot representation of IP-MS experiments using lysates from Myc-tagged Bdp1-expressing WT and *cdk1-as1* strains. Cells were synchronized in α factor and released into the cell cycle until they reached S phase. Bdp1-Myc was purified from lysates using magnetic anti-Myc-beads, after which copurifying proteins were analyzed by quantitative MS. (**B**) Metascape gene ontology analysis of the proteins showing a decreased interaction with Bdp1 in *cdk1-as1* mutants. Metascape settings: Min overlapping 7; *P* value 0.01; Min Enrichment 2. The size of the nodes indicates the relative number of proteins associated with that specific GO term; node color indicates the *P* value. (**C**) Cdk1 activity promotes the interaction between Bdp1 and Tfc4. Untagged WT cells, *BDP1-MYC TFC4-HA CDK1* cells, and *BDP1-MYC TFC4-HA cdk1-as1* mutants were synchronized with pheromone, released, and harvested in S phase. Bdp1 was immunoprecipitated from cell lysates using Myc antibodies, and analyzed by SDS-PAGE/western blotting (WB) using anti-Myc and anti-HA antibodies (*left panel*). Quantification of WBs from three independent experiments is shown in the bar chart (*right panel*). Asterisk, *P*<0.05. *P* values were calculated using Student's *t*-test. (**D**) Cdk1 increases Tfc4 levels at tRNA genes. WT cells or *cdk1-as1* mutants expressing *TFC4-HA* were synchronized with pheromone and the level of Tfc4 at the indicated *tDNAs* was analyzed by ChIP-qPCR. Values were normalized to *CDK1* at time-point zero after alpha factor release. *P* values were calculated using Student's *t*-test. Cell cycle analysis by flow cytometry is shown to the right. (**E**) Cdk1 promotes the interaction of Tfc4 with *tDNA*. S phase extracts of *RPO31-HA, BDP1-MYC, or TFC4-HA*-expressing cells, in a WT or in a *cdk1-as1* background, were incubated with either biotinylated control DNA or a *tDNA* template (both immobilized on magnetic beads) for 25 min at 30°C. Beads were washed with buffer containing 100, 200 or 350 mM NaCl, after which Rpo31-HA, Bdp1-Myc and Tfc4-HA proteins were eluted from the beads with Laemmli buffer and analyzed by WB. (**F**) Quantifications of the WBs shown in (E). Values are normalized to 100 mM NaCl wash of WT cells. Asterisk indicates significant difference (*P* < 0.05) between WT cells and *cdk1-as1* mutants. Error bars indicate SEM of three independent experiments. *P* values were calculated using Student's *t*-test.

To better understand how Cdk1 might control *tDNA* transcription at the molecular level, we studied the effect of Cdk1 on the composition of the RNAPIII and TFIIIB protein complexes. We analyzed quantitatively the interactions of Bdp1 (a subunit of TFIIIB) with other RNAPIII components in WT cells and in *cdk1-as1* mutants using immunoprecipitation followed by mass spectrometry (IP-MS (27)). Interestingly, the interactions between Bdp1 and other components of TFIIIB (i.e. Brf1 and TBP), as well as the τ^A^ subcomplex of TFIIIC (particularly Tfc4), were substantially reduced in the *cdk1-as1* mutant (Figure 4A and Supp Dataset 4). As expected, Metascape analysis revealed that these altered interactions primarily affected proteins involved in RNAPIII transcription (Figure 4B). These MS data were confirmed by co-IP experiments, which showed that the interaction between Bdp1 and Tfc4 was reduced by ∼40–50% in the *cdk1-as1* mutant compared with WT cells (Figure 4C). These data indicate that Cdk1 promotes the interaction between TFIIIB and TFIIIC.

Next, we analyzed the levels of Rpo31, Bdp1 and Tfc4 at *tRNA^iM^* and *tRNA^W^* in vivo by ChIP-qPCR using synchronized cell cultures. While there was no significant change in the recruitment of either Rpo31 or Bdp1 during the cell cycle (Supplementary Figure S2G and H), recruitment of Tfc4 peaked sharply in WT cells during S phase, and this did not occur in *cdk1-as1* mutant cells (Figure 4D). These data show that Cdk1 promotes recruitment of Tfc4 during S phase.

To study the effect of Cdk1 on binding of Tfc4, Bdp1 and Rpo31 to DNA, we performed an *in vitro tDNA* pull-down experiment in which native S phase cell extracts were incubated with either an immobilized *tDNA* template (*tL(CAA)C*) or with immobilized control DNA (see Materials and Methods). The TFIIIB-tDNA complex is stable as it resists dissociation by heparin and high salt concentrations, conditions under which TFIIIC is stripped from the DNA (13). Therefore, the pull-downs were washed with increasing salt concentrations to gradually destabilize the interactions between the complexes, followed by western blotting to analyze binding of Rpo31, Bdp1 and Tfc4 to the DNA templates. We found that all three proteins preferentially bound the *tDNA* template (Figure 4E, compare lane 1 with 2 and lane 5 with 6). Equal amounts of Rpo31 and Bdp1 were associated with the *tDNA* template in WT cells and *cdk1-as1* mutants, reflecting our ChIP-qPCR findings (Supplementary Figure S2G) However, we found that the level of *tDNA*-bound Tfc4 was slightly but significantly lower in *cdk1-as1* mutants at 100 mM NaCl (Figure 4E, lanes 2 and 6; quantified in Figure 4F), which is consistent with our ChIP-qPCR findings shown in Figure 4D. Washing with higher concentrations of NaCl effectively removed nearly all Tfc4 from the template, irrespective of Cdk1 activity (Figure 4E, lanes 4 and 8; quantified in Figure 4F). In contrast, the interaction of Bdp1 with the *tDNA* template was less sensitive to salt washing, and Bdp1 appeared to be more tightly associated with the *tDNA* template in *cdk1-as1* mutants than in WT cells (Figure 4E, compare lanes 4 and 8; quantified in Figure 4F).

Together, these data show that Cdk1 increases TFIIIC levels at *tDNA* during S phase, and suggest that Cdk1 may affect the turnover of TFIIIB and TFIIIC on chromatin, thereby affecting the dynamic interaction between TFIIIB, TFIIIC and RNAPIII.

### Bdp1 phosphorylation is required for increased tRNA expression in S phase

**Figure 5.**
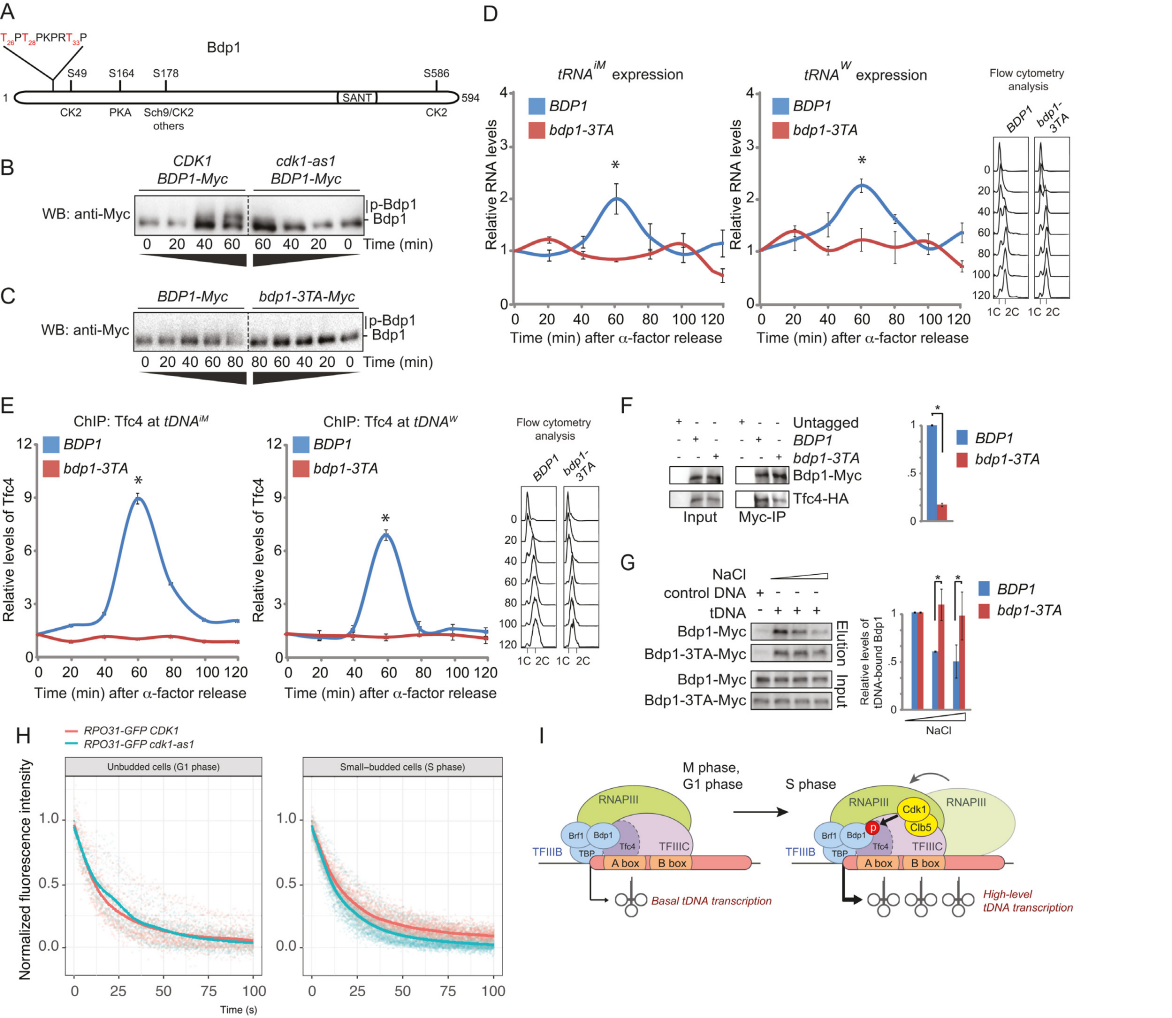
Bdp1 phosphorylation is required for the cell cycle-dependent peak in tRNA expression in S phase. (**A**) Protein domain organization and phosphorylation sites in Bpd1. Potential Cdk1 sites are shown in red. (**B**) Cell cycle-dependent Bdp1 phosphorylation depends on Cdk1 activity. Synchronized cell lysates were prepared as in Figure 1A and analyzed by Phos-tag SDS-PAGE and blotting with anti-Myc antibodies. (**C**) Diminished cell cycle-dependent phosphorylation of Bdp1 in *bdp1–3TA*-expressing cells. Cells expressing *BDP1-Myc* or *bdp1–3TA-Myc* were analyzed as described in (B). (**D**) Bdp1 phosphorylation contributes to cell cycle-dependent stimulation of *tDNA* transcription. *tRNA^iM^* and immature *tRNA^W^* expression levels in WT cells and *bdp1–3TA* mutants were analyzed as in Figure 1B. Values were normalized to WT at time-point zero. Error bars, SEM of three independent experiments. Cell cycle analysis is shown to the right. (**E**) Cell cycle-dependent recruitment of Tfc4 involves phosphorylation of Bdp1. Tfc4 levels were analyzed at the indicated *tDNA* genes as described in Figure 1A in either WT cells or in *bdp1–3TA* mutants. Values were normalized to WT at time-point zero. Cell cycle analysis is shown to the right. (**F**) Tfc4 binds more efficiently to WT Bdp1 than to non-phosphorylatable Bdp1–3TA. WT cells and *bdp1–3TA* mutants were collected in S phase and the interaction between Bdp1 and Tfc4 was analyzed as described in Figure 4C. Quantification of the WB is shown in the bar chart to the right. Error bars indicate SEM of three independent experiments. Asterisk, *P* < 0.05. *P* values were calculated using Student's *t*-test. (**G**) *bdp1–3TA* binds more stably to *tDNA. Left panel: In vitro tDNA* pull-down experiments using S phase extracts were performed as described in Figure 4E. *Right panel:* Quantification of the western blot experiments. Values are normalized to 100 mM NaCl wash of wild-type Bdp1-Myc. Error bars indicate SEM of three independent experiments. Asterisk, *P* < 0.05. *P* values were calculated using Student's *t*-test. (**H**) Analysis of relative Rpo31 dynamics by FLIP. 50% of the nucleus was bleached every 500 ms for 100 s, and the fluorescence loss of GFP in the unbleached half of the nucleus was monitored. Curves represent smoothed averages of all data points. Number of cells imaged: *RPO31-GFP CDK1*, G1 phase (*n* = 13); *RPO31-GFP CDK1*, S phase (*n* = 21); *RPO31-GPF cdk1-as1*, G1 phase (*n* = 9); *RPO31-GPF cdk1-as1*, S phase (*n* = 28). (**I**) Model for cell cycle-induced stimulation of *tDNA* transcription. While basal *tDNA* transcription takes place during most or all of the cell cycle, it peaks during S phase due to recruitment of Clb5-Cdk1. Cdk1-dependent phosphorylation of Bdp1 promotes Tfc4 recruitment, leading to enhanced RNAPIII turnover and increased *tDNA* transcription.

Yeast Bdp1 has been shown to be phosphorylated by PKA, Sch9 and CK2 on four major residues, resulting in increased RNAPIII transcriptional activity (29). In addition, several phosphoproteomics studies have found that Bdp1 may also be phosphorylated on at least two potential Cdk1 sites, i.e. T26 and T33 (Figure 5A) (48,49). Interestingly, we found that Bdp1 phosphorylation increased when cells were released from pheromone-induced cell cycle arrest, but this was diminished in cells expressing *cdk1-as1* (Figure 5B), indicating that Cdk1 activity contributes to phosphorylation of Bdp1. We generated a mutant allele of *BDP1, bdp1-3TA*, in which we alanine-substituted T26 and T33, in addition to a third potential Cdk1 site located in between these two sites (T28), and found that mutation of these sites substantially reduced the cell cycle-dependent phosphorylation of Bdp1 (Figure 5C). We used this mutant to study *tDNA* transcription during the cell cycle. Interestingly, the peak in *tDNA* transcription that normally occurs in S phase was no longer observed in cells that express *bdp1-3TA* as the sole source of Bdp1 (Figure 5D). This correlated with a substantial reduction in recruitment of Tfc4 to *tDNA* genes in *bdp1-3TA* mutants compared to cells expressing wild-type *BDP1* (Figure 5E), indicating that phosphorylation of Bdp1 may be important for efficient recruitment of Tfc4. Indeed, less Tfc4 coimmunoprecipitated with Bdp1-3TA than with wild-type Bdp1 (Figure 5F), mirroring our finding that the interaction between Bdp1 and Tfc4 is weaker in *cdk1-as1* mutants than in WT cells (Figure 4C). Furthermore, *in vitro* pulldown experiments showed that Bdp1-3TA bound more stably to a *tDNA* template than normal Bdp1 (Figure 5G), which is also consistent with experiments performed with *cdk1-as1* mutants (Figure 4E), and suggest that phosphorylation of Bdp1 promotes its turnover on the DNA. Together, these data indicate that phosphorylation of Bdp1 contributes to the temporary increase in *tDNA* transcription during S phase.

### Cdk1 restricts nuclear diffusion of RNAPIII during S phase

Finally, given that rapid, repetitive cycles of RNAPIII initiation underlie high transcription levels (50), we wished to gain insight in the effect of Cdk1 on RNAPIII dynamics in single, living cells. Our ChIP-qPCR experiments and *in vitro* pulldown data indicate that Cdk1 does not promote *tDNA* transcription by increasing the overall levels of RNAPIII to *tDNA*. However, these steady-state assays only present a snapshot of RNAPIII association with *tDNA* and fail to capture the dynamic behavior of proteins on chromatin in living cells. Furthermore, ChIP-qPCR merely provides an estimate of the relative levels of a protein at a given genomic site in an averaged pool of cells, which may not accurately reflect the actual occupancy and behavior of that protein in individual cells. Therefore, we made use of fluorescence loss in photobleaching (FLIP), which is a commonly used single-cell imaging method to study the dynamic behavior of proteins in living cells (51). Briefly, we monitored Rpo31-GFP dynamics in WT and *cdk1-as1* cells by determining signal decay of fluorescence during photobleaching. Half of the nucleus was bleached every 500 ms, while loss of fluorescence was measured in the other half of the nucleus, after which we determined the decay constant (which reflects residency time) for both strains. G1 phase and S phase cells were identified by their morphology; in *S. cerevisiae*, G1 phase cells are unbudded, whereas S phase cells have small buds (52). Interestingly, in WT cells Rpo31-GFP fluorescence decayed slower in S phase cells than in G1 cells, suggesting that a larger proportion of Rpo31 is immobilized on chromatin in S phase compared to G1 phase (Figure 5H and Supplementary Figure S2I). In contrast, in *cdk1-as1* mutant cells the decay in fluorescence was indistinguishable between G1 and in S phase cells (Figure 5H and Supplementary Figure S2I), indicating that this mutant failed to increase Rpo31 retention on chromatin during S phase. These data suggest that Cdk1 activity reduces the ability of Rpo31 to diffuse through the nucleus, which is consistent with a model in which Cdk1 promotes RNAPIII reinitiation on tRNA genes during S phase to stimulate *tDNA* transcription.

## DISCUSSION

Here, we have shown that Cdk1 induces a brief cell cycle-dependent increase in *tDNA* transcription. Using ChIP-seq we found that Cdk1 is present at all tRNA genes. One potential pitfall of ChIPseq is that certain areas of the genome, including *tDNA*, are prone to be aspecifically enriched by this method, often referred to as hyperChIPable regions (53). However, as a control, free GFP could not be detected at tRNA genes, and localization of Cdk1 to *tDNA* occurs only periodically during the cell cycle and specifically requires *CLB5*. Furthermore, only Clb5-GFP (and to a much lesser extent Clb6-GFP), but none of the other GFP-tagged cyclins, could be detected at *tDNA*. These findings are incompatible with the hyperChIPable artefact hypothesis, and we conclude that recruitment of Cdk1 is specific. While Clb5 appears to be the main cyclin responsible for Cdk1 recruitment, and both Clb5 and Cdk1 activity are required for increased tRNA synthesis during S phase, Clb6 also slightly contributes to the cell cycle-stimulated increase in tRNA synthesis. This is consistent with the fact that *CLB6* is only expressed very briefly during the cell cycle, whereas Clb5 is stable until mitosis (54), when it is degraded by the Anaphase Promoting Complex (APC, (55)). Exactly how the Clb5-Cdk1 complex is recruited to *tDNA* remains to be established. A high-throughput proteomics study has found several physical interactions between Cdk1 and RNAPIII (56), whereas a recent low-throughput study also found that Cdk1 physically interacts with the TFIIIC component Tfc7 (57), suggesting that Clb5-Cdk1 may be recruited to *tDNA* by TFIIIC, which is the focus of our ongoing studies. Genetic support for a link between Cdk1, tRNA synthesis and cell cycle progression comes from studies that identified multiple negative genetic interactions between genes that encode components of the Cdk1 holoenzyme and genes required for *tDNA* transcription and maturation (Supplementary Figure S2J) (58). For instance, mutations in either *CDK1* or in *CKS1*, which encodes an adaptor protein important for activity of the Cdk1 holoenzyme, result in synthetic lethality when combined with hypomorphic mutations in *BDP1* and *TFC3* (which encodes a component of TFIIIC) (58).

A previous study has also found that tRNA synthesis fluctuates during the cell cycle in *S. cerevisiae*, although it was shown that *tDNA* transcription peaked in M phase rather than S phase (30). One explanation for this apparent discrepancy is that S phase overlaps with metaphase in *S. cerevisiae* (40), making it difficult to discern between these two stages of the cell cycle. The increase in *tDNA* transcription was almost completely abolished in mutants lacking the S phase cyclin Clb5 (and to a lesser extent Clb6). Mutants lacking cyclins that regulate later stages of the cell cycle (Clb1–4 (1)), particularly the main M phase cyclin Clb2, had no defect at all (Supplementary Figure S2F). Since Clb5 is degraded by Cdc20 in metaphase, and because of the overlap between metaphase and S phase, we conclude that the cell cycle-dependent increase in *tDNA* transcription occurs in the overlapping time span of late S phase/early metaphase.

Cell cycle regulation of RNAPIII activity appears to be evolutionarily conserved, and our findings that tRNA synthesis increases in S phase are supported by an early study in mammalian cells, which showed that tRNA synthesis is low in M and G1 phase and peaks in S and G2 phase (59). TFIIIB appears to be an important target for cell cycle regulation of RNAPIII, although the underlying mechanisms appear to have diverged between yeast and more complex eukaryotes. For instance, while we found that Clb5-Cdk1 activates RNAPIII in S phase, addition of purified Cyclin B1-Cdk1 complexes to *Xenopus laevis* egg extracts represses TFIIIB to inhibit RNAPIII activity in M phase (60). RNAPIII activity is also inhibited in M phase in mammalian cells (59,61), and this mitotic repression requires CK2 rather than Cdk1 (62). These differences between yeast and more complex organisms may in part be due to the fact that complex eukaryotes generally repress transcription in M phase (63,64), which is much less pronounced in *S. cerevisiae* (65).

We identified the TFIIIB component Bdp1 as a potential cell cycle-dependent target of Cdk1. At present, we cannot rule out the possibility that additional Cdk1 targets exist that regulate tRNA synthesis, that Bdp1 phosphorylation is mediated by other proline-directed kinases downstream of Cdk1, or that Bdp1 phosphorylation serves additional purposes, such as formation of chromatin boundaries. This is the focus of our ongoing studies. The potential Cdk1 sites in Bdp1 (T26, T28 and T33) are different from previously identified, major phosphorylation sites (S49, S164, S178 and S586), which are targeted by PKA, Sch9 and CK2 under optimal nutrient levels (29). Interestingly, phosphorylation of these four residues also stimulates TFIIIB activity, suggesting that Bdp1 is a common target for pathways that promote cell growth and proliferation (29).

Preventing Bdp1 phosphorylation resulted in reduced binding of Bdp1 to Tfc4 and loss of the cell cycle-dependent increase in Tfc4 levels at tRNA genes. This stimulatory effect of Bdp1 on the recruitment of Tfc4 during S phase was surprising, because TFIIIC is generally believed to function upstream of TFIIIB, promoting assembly of TFIIIB at tRNA genes, and TFIIIB alone is sufficient to drive transcription *in vitro* (50). However, it has been shown that TFIIIC contributes to high-efficiency transcription by promoting recapture of RNAPIII, which is particularly apparent on longer transcription units (17), although it likely also occurs on *tDNA* templates (19). Furthermore, TFIIIC may promote reinitiation of RNAPIII on the same *tDNA* template by increasing the efficiency of transcription termination (15,16). TFIIIC has also been reported to establish a gene loop to facilitate transcription reinitiation (66). Our findings are consistent with a previous model in which phosphorylation of Bdp1 favors facilitated recycling of RNAPIII on the *tDNA* template (29). We believe that Cdk1-dependent phosphorylation of Bdp1 may contribute to this process by promoting the interaction between TFIIIB and TFIIIC, thereby temporarily increasing the level of TFIIIC at tRNA genes during the cell cycle (Figure 5I). As a consequence, this may enhance the dynamics of *tDNA* transcription, e.g. by increasing the turnover of basal transcription factors or RNAPIII on the *tDNA* template, by increasing the efficiency of termination, or by stabilizing a gene loop. This process may not be easily reconstituted in *in vitro* assays with purified proteins, because it involves post-translational modifications at specific time intervals. Further studies are required to dissect the exact molecular mechanism of this process.

What is the physiological relevance of the cell cycle-induced increase in tRNA synthesis? The volume of daughter cells at birth is ∼30 fL, increasing to ∼57 fl at the time of cell division (40 and 74 fl, respectively, for mother cells that have undergone previous cell divisions (67)). Thus, a cell's volume increases nearly two-fold between birth and cell division, during which the tRNA pool is divided between mother and daughter cells, both of whom subsequently increase their volume as they start a new round of budding. This means that during the cell cycle the tRNA pool can fluctuate substantially, although this is normally not observed in WT cells because they can compensate for the dilution of the tRNA pool by increasing tRNA synthesis during S phase (as reported in this manuscript). However, mutants that fail to synchronize tRNA synthesis with cell growth, such as *cdk1* mutants, have lower levels of tRNA overall (both mature and immature). Bulk bud growth takes place during late S phase (68), and therefore *tDNA* transcription should be higher during this phase of the cell cycle, which is exactly what we observed. Our findings are consistent with our previous publication in which we showed that Cdk1 localizes to hundreds of highly expressed, RNAPII-transcribed genes, particularly housekeeping genes, to boost their transcription during cell division (2), suggesting that Cdk1 helps set the transcriptional amplitude of genes required for cell growth and homeostasis. Cdk1 has long been known to promote protein synthesis (69,70), and here we have shown that Cdk1 promotes *tDNA* transcription, indicating that Cdk1 coordinates tRNA synthesis with the translational and anabolic needs of the cell during cell division.

## CONCLUSIONS

How cells regulate RNAPIII is still very poorly understood. This study revealed a potential molecular mechanism by which RNAPIII activity is controlled, and identified a novel, direct connection between tRNA synthesis and the cell cycle.

## DATA AVAILABILITY

The RNAseq data have been deposited to GEO with the dataset identifier GSE116224. The mass spectrometry proteomics data have been deposited to the ProteomeXchange Consortium via the PRIDE partner repository with the dataset identifier PXD011084.

## Supplementary Material

Supplementary Data
