# Cdk1 gates cell cycle-dependent tRNA synthesis by regulating RNA polymerase III activity

**DOI:** 10.1093/nar/gky1102

**Published:** 2018-11-05

**Authors:** Maria C Herrera, Pierre Chymkowitch, Joseph M Robertson, Jens Eriksson, Stig Ove Bøe, Ingrun Alseth, Jorrit M Enserink

**Affiliations:** 1 Department of Molecular Cell Biology, Institute for Cancer Research, the Norwegian Radium Hospital, Montebello, N-0379 Oslo, Norway; 2 Centre for Cancer Cell Reprogramming, Institute of Clinical Medicine, Faculty of Medicine, University of Oslo, Oslo, Norway; 3 The Department of Biosciences, Faculty of Mathematics and Natural Sciences, University of Oslo, 0371, Norway; 4 Department of Medical Biochemistry, Oslo University Hospital, Oslo, Norway; 5 Department of Microbiology, Oslo University Hospital, Oslo, Norway

*Nucleic Acids Research*,2018, https://doi.org/10.1093/nar/gky846

The authors wish to correct an error in their article. During preparation of the revised manuscript, an older temporary version of Figure 4 was submitted by accident. Figure 4E and quantifications in Figure 4F are incorrect. The description of Figure 4F in the text is also inaccurate and should read (changes are in bold): ‘In contrast, the interaction of Bdp1 with the tDNA template was less sensitive to salt washing, and Bdp1 appeared to be more tightly associated with the tDNA template in cdk1-as1 mutants than in WT cells (Figure 4E, compare lanes **2, 3 and** 4 and lanes **6, 7 and** 8; quantified in Figure 4F).’

**Figure 4.**
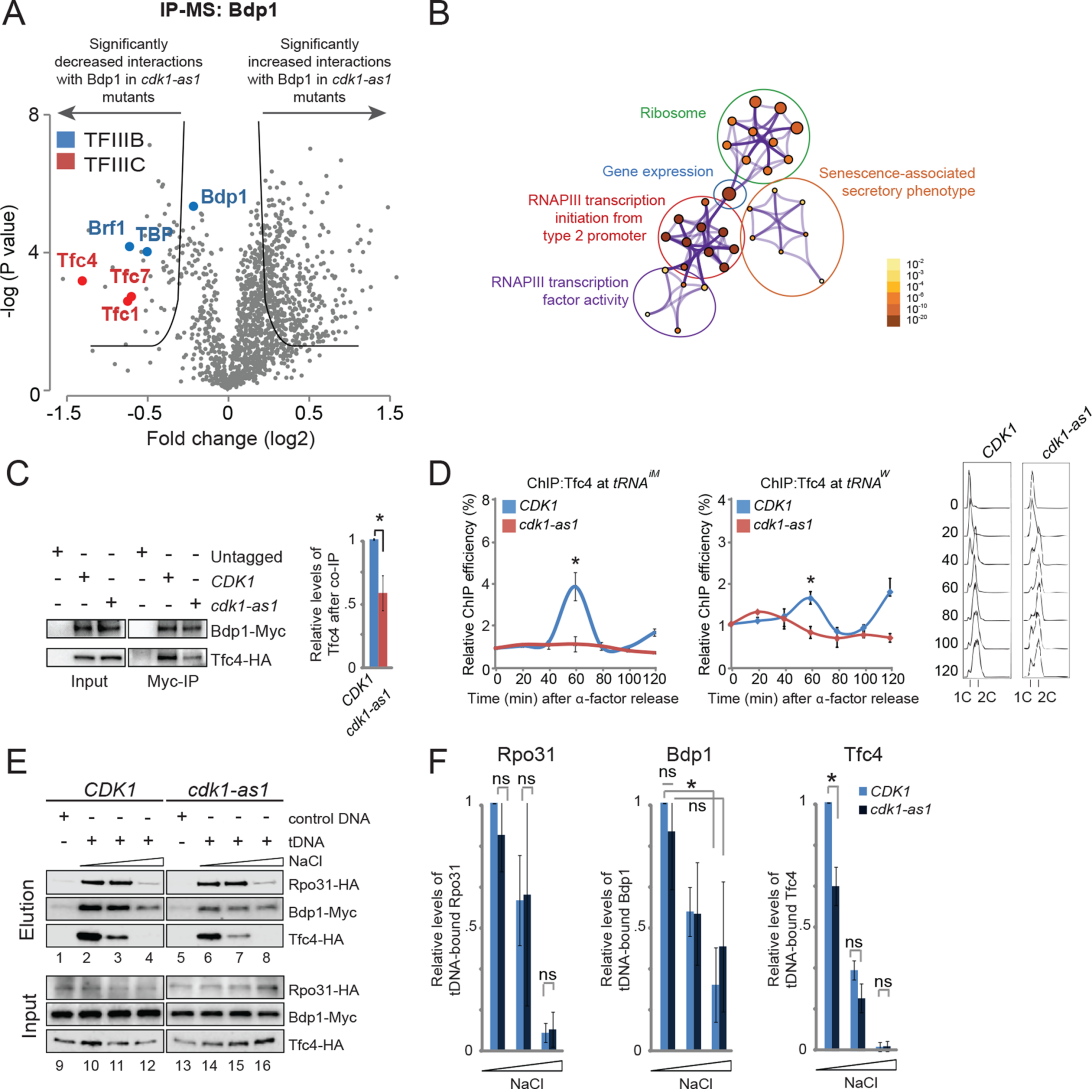
Cdk1 promotes the interaction between Bdp1 and Tfc4. (**A**) Volcano plot representation of IP-MS experiments using lysates from Myc-tagged Bdp1-expressing WT and *cdk1-as1* strains. Cells were synchronized in α factor and released into the cell cycle until they reached S phase. Bdp1-Myc was purified from lysates using magnetic anti-Myc-beads, after which copurifying proteins were analyzed by quantitative MS. (**B**) Metascape gene ontology analysis of the proteins showing a decreased interaction with Bdp1 in *cdk1-as1* mutants. Metascape settings: Min overlapping 7; *P* value 0.01; Min Enrichment 2. The size of the nodes indicates the relative number of proteins associated with that specific GO term; node color indicates the *P* value. (**C**) Cdk1 activity promotes the interaction between Bdp1 and Tfc4. Untagged WT cells, *BDP1-MYC TFC4-HA CDK1* cells, and *BDP1-MYC TFC4-HA cdk1-as1* mutants were synchronized with pheromone, released, and harvested in S phase. Bdp1 was immunoprecipitated from cell lysates using Myc antibodies, and analyzed by SDS-PAGE/western blotting (WB) using anti-Myc and anti-HA antibodies (*left panel*). Quantification of WBs from three independent experiments is shown in the bar chart (*right panel*). Asterisk, *P*<0.05. *P* values were calculated using Student's *t*-test. (**D**) Cdk1 increases Tfc4 levels at tRNA genes. WT cells or *cdk1-as1* mutants expressing *TFC4-HA* were synchronized with pheromone and the level of Tfc4 at the indicated *tDNAs* was analyzed by ChIP-qPCR. Values were normalized to *CDK1* at time-point zero after alpha factor release. *P* values were calculated using Student's *t*-test. Cell cycle analysis by flow cytometry is shown to the right. (**E**) Cdk1 promotes the interaction of Tfc4 with *tDNA*. S phase extracts of *RPO31-HA, BDP1-MYC, or TFC4-HA*-expressing cells, in a WT or in a *cdk1-as1* background, were incubated with either biotinylated control DNA or a *tDNA* template (both immobilized on magnetic beads) for 25 min at 30°C. Beads were washed with buffer containing 100, 200 or 350 mM NaCl, after which Rpo31-HA, Bdp1-Myc and Tfc4-HA proteins were eluted from the beads with Laemmli buffer and analyzed by WB. (**F**) Quantifications of the WBs shown in (E). Values are normalized to 100 mM NaCl wash of WT cells. Asterisk indicates significant difference (*P* < 0.05) between WT cells and *cdk1-as1* mutants. Error bars indicate SEM of three independent experiments. *P* values were calculated using Student's *t*-test.

The correct version of Figure 4 is provided below.

While the corrected figure looks slightly different, these corrections do not alter the conclusions of the experiments, which are that binding of Bdp1 to tDNA is more stable in cdk1-as1 mutants than in WT cells.

The article has been updated online and in print.

