# Supplementary material for: Cdk1 gates cell cycle-dependent tRNA synthesis by regulating RNA polymerase III activity
Source: Nucleic Acids Res. 2018 Sep 22;46(22):11698–711. doi: 10.1093/nar/gky846 (PMC6294503; doi:10.1093/nar/gky846)
Supplement: Supplementary Data [file gky846_supplemental_files.zip › Suppl.-Table-S2.docx]

**Supplemental Table S2.** Primers used in this study.

| ChIP-qPCR | |
| --- | --- |
| Target/ID | Sequence |
| *tDNA(iM)/*  853501 | GAAGCGCGCAGGGCTCATAA |
|  | TAGCGCCGCTCGGTTTCGAT |
| *tDNA(W)/*  853103 | GGAATTTCCAAGATTTAATTGGAGTCG |
|  | TGGCTCAATGGTAGAGCTTTCG |
| *PMA1/*  852876 | GATCCACCAAGAGACGATACTGCT |
|  | ACCGCCACCTAGACCTAATCTTTC |
| *SSE1/*  855998 | ACCTCGATAGCCATAAGCAAA |
|  | GCAACGGCAAGGACAGAGTTATTG |
| Expression analysis (RT-qPCR) | |
| Target | Sequence |
| *tDNA(iM)/*  853501 | GAAGCGCGCAGGGCTCATAA |
|  | TAGCGCCGCTCGGTTTCGAT |
| *tDNA(W)/*  853103 | GGAATTTCCAAGATTTAATTGGAGTCG |
|  | TGGCTCAATGGTAGAGCTTTCG |
| Northern analysis | |
| Target | Sequence |
| *tDNA(iM)* | TAGCGCCGCTCGGTTTCGAT |
| *tDNA(W)* | CAGGAATTGAACCTGCAACCCTTC |
| *tDNA(L)* | GGTTGCTAAGAGATTCGAACTC |
| *In vitro* tDNA pulldown | |
| Target | Sequence |
| *tDNA(Leu3)/*  852843 | GTACTTCCTTGTTCATGTGTGTTC |
|  | Bt-CCCTGTGTGTTCTCGTTATGT |
| *pLVX-braf* | GATGGCACCAGAAGTCATCAG |
|  | Bt-GAAGCTGCGCCTGTCTTAG |
