# Supplementary material for: Cdk1 gates cell cycle-dependent tRNA synthesis by regulating RNA polymerase III activity
Source: Nucleic Acids Res. 2018 Sep 22;46(22):11698–711. doi: 10.1093/nar/gky846 (PMC6294503; doi:10.1093/nar/gky846)
Supplement: Supplementary Data [file gky846_supplemental_files.zip › Suppl._Table_S1_strains -.docx]

**Supplemental Table S1**

**Strains**:

| Strain no. | Relevant genotype | Source |
| --- | --- | --- |
| BY4741 | MATa, his3Δ1, leu2Δ0, met15Δ, ura3Δ0 | [1] |
| RDKY3023 | MATa, ura3-52, leu2Δ1, trp1Δ63, his3-200, lys2-Bgl, hom3-10, ade2Δ1, ade8 | [2] |
| JEY5340 | *BY4741 CDC28-TAP:HIS3MX6* | [3] |
| JEY9109 | MATa, his3Δ1, leu2Δ0, met15Δ, ura3Δ0, CDC28-GFP:HIS3MX6 | [4] |
| JEY251 | MATa, ura3-52, leu2Δ1, trp1Δ63, his3-200, lys2-Bgl, hom3-10, ade2Δ1, ade8, yel069::URA3, cdc28-as1:TRP1 | [5] |
| JEY499 | MATa, ura3-52, leu2Δ1, trp1Δ63, his3-200, lys2-Bgl, hom3-10, ade2Δ1, ade8, yel069::URA3, cdc28-5M:TRP1 | [5] |
| JEY6198 | MATa, ura3-52, leu2Δ1, trp1Δ63, his3-200, lys2-Bgl, hom3-10, ade2Δ1, ade8, CLB5-9MYC:KANMX6 | This study |
| JEY6294 | MATa, ura3-52, leu2Δ1, trp1Δ63, his3-200, lys2-Bgl, hom3-10, ade2Δ1, ade8, CLB6-6HA:KANMX6 | This study |
| JEY11208 | MATa, his3Δ1, leu2Δ0, met15Δ, ura3Δ0, CDC28-GFP:HIS3MX6 clb5*::*natNT2 | This study |
| BY4741 | MATa, his3Δ1, leu2Δ0, met15Δ, ura3Δ0, clb5 Δ | This study |
| JEY8300 | MATa, ura3-52, leu2Δ1, trp1Δ63, his3-200, lys2-Bgl, hom3-10, ade2Δ1, ade8, maf1::hph | This study |
| JEY8303 | MATa, ura3-52, leu2Δ1, trp1Δ63, his3-200, lys2-Bgl, hom3-10, ade2Δ1, ade8, maf1::hph, cdc28-as1:TRP1 | This study |
| JEY8620 | MATa, ura3-52, leu2Δ1, trp1Δ63, his3-200, lys2-Bgl, hom3-10, ade2Δ1, ade8, YEL069C, RPO31-6HA:natNT2 | This study |
| JEY8624 | MATa, ura3-52, leu2Δ1, trp1Δ63, his3-200, lys2-Bgl, hom3-10, ade2Δ1, ade8, YEL069C, RPO31-6HA:NAT, cdc28-as1:TRP1 | This study |
| JEY8331 | MATa, ura3-52, leu2Δ1, trp1Δ63, his3-200, lys2-Bgl, hom3-10, ade2Δ1, ade8, BDP1-9MYC:KANMX | This study |
| JEY8335 | MATa, ura3-52, leu2Δ1, trp1Δ63, his3-200, lys2-Bgl, hom3-10, ade2Δ1, ade8, BDP1-9MYC:KANMX, cdc28-as1:TRP1 | This study |
| JEY11562 | MATa *,ura3-52, leu2∆1, trp1∆63, his3∆200, lys2∆Bgl, hom3-10, ade2∆1, ade8, TFC4-6HA:hphNT1* | This study |
| JEY11564 | MATa *,ura3-52, leu2∆1, trp1∆63, his3∆200, lys2∆Bgl, hom3-10, ade2∆1, ade8, TFC4-6HA:hphNT1, yel069::URA3, cdc28-as1:TRP1* | This study |
| JEY11995 | MATa *,ura3-52, leu2∆1, trp1∆63, his3∆200, lys2∆Bgl, hom3-10, ade2∆1, ade8,* BDP1-9MYC:KANMX *, TFC4-6HA:hphNT1* | This study |
| JEY11999 | MATa *,ura3-52, leu2∆1, trp1∆63, his3∆200, lys2∆Bgl, hom3-10, ade2∆1, ade8,* BDP1-9MYC:KANMX *, TFC4-6HA:hphNT1,* cdc28-as1:TRP1 | This study |
| JEY11977 | MATa, his3Δ1, leu2Δ0, met15Δ, ura3Δ0, *GPD__pr_-natNT2:BDP1* | This study |
| JEY11978 | MATa, his3Δ1, leu2Δ0, met15Δ, ura3Δ0, *GPD__pr_-natNT2:bdp1-3TA* | This study |
| JEY11982 | MATa, *ura3-52, leu2∆1, trp1∆63, his3∆200, lys2∆Bgl, hom3-10, ade2∆1, ade8, GPD__pr_-natNT2:BDP1-9MYC:KANMX* | This study |
| JEY11983 | MATa, *ura3-52, leu2∆1, trp1∆63, his3∆200, lys2∆Bgl, hom3-10, ade2∆1, ade8, GPD__pr_-natNT2:bdp1-3TA-9MYC:KANMX)* | This study |
| JEY12120 | MATa *,ura3-52, leu2∆1, trp1∆63, his3∆200, lys2∆Bgl, hom3-10, ade2∆1, ade8, TFC4-6HA:HIS3MX6, GPD__pr_-natNT2:*BDP1-9MYC:KANMX | This study |
| JEY12121 | MATa *,ura3-52, leu2∆1, trp1∆63, his3∆200, lys2∆Bgl, hom3-10, ade2∆1, ade8, TFC4-6HA:HIS3MX6, GPD__pr_-natNT2:bdp1-3TA* -9MYC:KANMX | This study |
| JEY9226 | MATa *,* his3Δ1, leu2Δ0, met15Δ, ura3Δ0, RPO31-GFP:HIS3MX6 | This study |
| JEY11985 | MATa *,* his3Δ1, leu2Δ0, met15Δ, ura3Δ0, RPO31-GFP:HIS3MX6, cdc28-as1:TRP1 | This study |
| JEY10870 | MATa, his3Δ1, leu2Δ0, met15Δ, ura3Δ0, CLB1-GFP:HIS3MX6 | This study |
| JEY10872 | MATa *,* his3Δ1, leu2Δ0, met15Δ, ura3Δ0, CLB3-GFP:HIS3MX6 | This study |
| JEY10869 | MATa *,* his3Δ1, leu2Δ0, met15Δ, ura3Δ0, CLN1-GFP:HIS3MX6 | This study |
| JEY10871 | MATa *,* his3Δ1, leu2Δ0, met15Δ, ura3Δ0, CLB2-GFP:HIS3MX6 | This study |
| JEY10873 | MATa *,* his3Δ1, leu2Δ0, met15Δ, ura3Δ0, CLB4-GFP:HIS3MX6 | This study |

**Plasmids**:

| *pBS-SK-tRNA^LEU3^ Amp^R^* | [6] |
| --- | --- |
| *pLVX-braf-1857–2302 Amp^R^* | This study |

**References**

1. Brachmann CB, Davies A, Cost GJ, Caputo E, Li J, Hieter P, Boeke JD. Designer deletion strains derived from Saccharomyces cerevisiae S288C: a useful set of strains and plasmids for PCR-mediated gene disruption and other applications. Yeast. 1998;14: 115-132.
2. Chen C, Kolodner RD. Gross chromosomal rearrangements in Saccharomyces cerevisiae replication and recombination defective mutants. Nat Genet. 1999;23: 81-85.
3. Ghaemmaghami S, Huh WK, Bower K, Howson RW, Belle A, Dephoure N, O'Shea EK, Weissman JS. Global analysis of protein expression in yeast. Nature. 2003;425: 737-741.
4. Huh WK, Falvo JV, Gerke LC, Carroll AS, Howson RW, Weissman JS, O'Shea EK. Global analysis of protein localization in budding yeast. Nature. 2003;425: 686-691.
5. Enserink JM, Hombauer H, Huang ME, Kolodner RD. Cdc28/Cdk1 positively and negatively affects genome stability in S. cerevisiae. J Cell Biol. 2009;185: 423-437.
6. Fan X, Shi H, Lis JT. Distinct transcriptional responses of RNA polymerases I, II and III to aptamers that bind TBP. Nucleic Acids Res. 2005;33: 838-845.
