# Supplementary material for: Cdk1 gates cell cycle-dependent tRNA synthesis by regulating RNA polymerase III activity
Source: Nucleic Acids Res. 2018 Sep 22;46(22):11698–711. doi: 10.1093/nar/gky846 (PMC6294503; doi:10.1093/nar/gky846)
Supplement: Supplementary Data [file gky846_supplemental_files.zip › Supplemental Figure Legends.docx]

**Supplemental Figure Legends**

**Suppl. Figure S1.** **Cdk1 is recruited to RNAPIII-transcribed genes**. ***A***, Normalized Cdk1 ChIP-seq binding regions are displayed for all *tDNAs.* Untagged (*CDK1*) and TAP-tagged Cdk1 (*CDK1-TAP*) strains were grown to log phase, lysed and analyzed by ChIP-seq using TAP antibodies. Data are derived from ([2](#_ENREF_2)). The y-axis scale represents normalized read density *CDK1-TAP* to *CDK1*. Mitochondrial *tDNAs* were excluded due to the SacCer3 genome reference used for the analysis. ***B***. Normalized Cdk1 ChIP-seq binding regions for non-tRNA class III genes (left); non-class III genes that are not regulated by Cdk1 (middle); and RNAPII-transcribed Cdk1 target genes (right). Data are derived from ([2](#_ENREF_2)). ***C***, Validation of ChIP-seq data by ChIP-qPCR. ChIP-qPCRs were performed on untagged (*CDK1)* and GFP-tagged (*CDK1-GFP*) strains using GFP antibodies and primers against *tRNA^iM^*, *tRNA^W^*, *PMA1*, *SSE1* and *ChrV*. Values are given as percentage of input. Error bars indicate SEM of three independent experiments. Asterisk, *p* < 0.05. NS, not significant. All *p* values were calculated using Student’s *t*-test.

**Supplemental Figure S2.** ***A,*** Cdk1 levels at *SSE1* are very low and do not change during the cell cycle. Cdk1 levels were analyzed at *SSE1* as described in Fig. 1A in either *CDK1* cells or in *CDK1-GFP* cells. ***B***, Analysis of *SSE1* expression levels during the cell cycle. WT *(CDK1)*, *cdk1-as1* and *cdk1-5M* strains were synchronized and sampled as in Fig. 1B and RNA levels were analyzed by RT-qPCR. Error bars indicate SEM of three independent experiments. All *p* values were calculated using Student’s *t*-test. ***C***, Cdk1-GFP and Rub1-GFP (control) levels at *tDNA^W^* in G1 and S phase were monitored by qPCR. Values are given as percentage of input and normalized to G1 phase for each strain Error bars indicate SEM of three independent experiments. Asterisk, *p* < 0.05, NS, not significant. ***D, E,*** *SNR52* (D) and *SCR1* (E) expression levels in *CDK1* and *cdk1-as1* cells in G1 and S phase. RNA levels were analyzed by RT-qPCR. Values were normalized to *CDK1* cells in G1 phase. Error bars indicate SEM of three independent experiments. Asterisk, *p* < 0.05. NS, not significant. All *p* values were calculated using Student’s *t*-test. ***F,*** Clb1, Clb3, Cln1, Clb2 and Clb4 are not recruited to *tDNA*. ChIP-qPCR assays were performed on an untagged control strain or on strains expressing GFP-tagged *CDK1, CLB1, CLB3, CLN1, CLB2* or *CLB4* using GFP antibodies and primer pairs against *tRNA^iM^*, *tRNA^W^* and *SSE1*. Values are given as percentage of input. Error bars indicate SEM of three independent experiments. Asterisk, *p*<0.05. NS, not significant. ***G, H,*** Rpo31 and Bdp1 steady-state levels at tRNA genes do not change during the cell cycle. Samples were processed and analyzed as described in Fig. 4D. ***I,*** The same graph as shown in Fig. 5H, but showing 99% confidence intervals (shaded areas). ***J,*** The CDK holoenzyme subunits Cdk1 and Cks1 physically (orange edges) and genetically (grey edges) interact with TFIIIB, TFIIIC, and various components of the RNAPIII machinery.
