# Supplementary figures and images for: Cdk1 gates cell cycle-dependent tRNA synthesis by regulating RNA polymerase III activity
Source: Nucleic Acids Res. 2018 Sep 22;46(22):11698–711. doi: 10.1093/nar/gky846 (PMC6294503; doi:10.1093/nar/gky846)

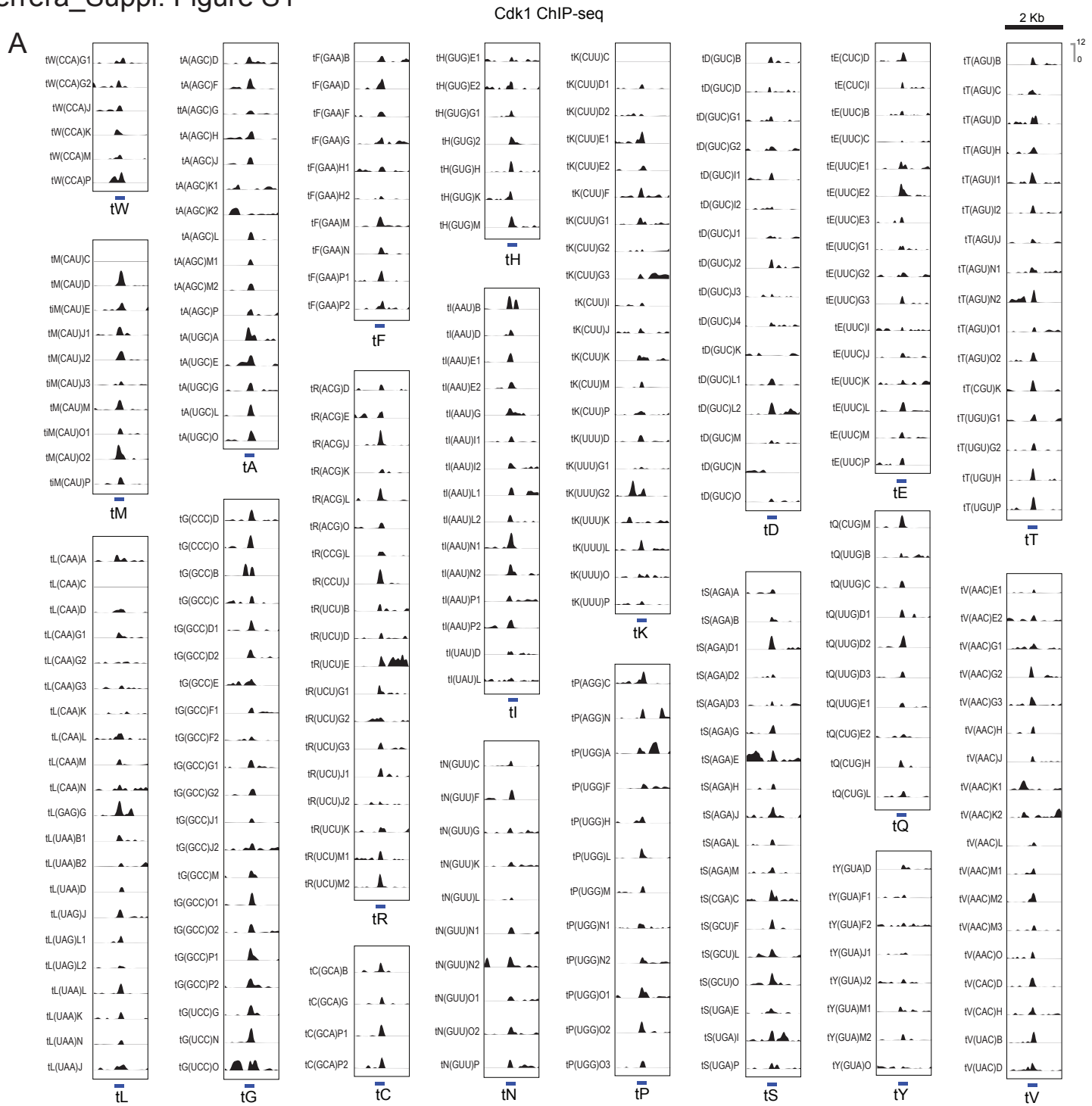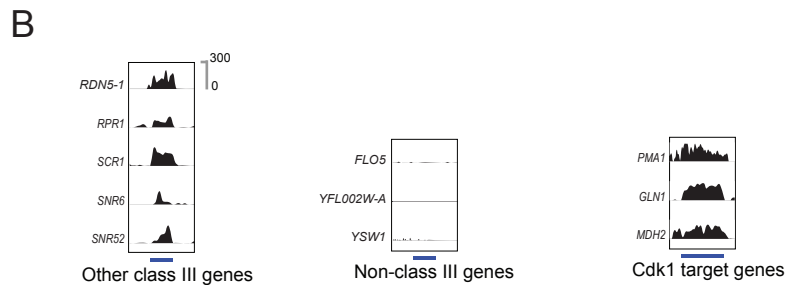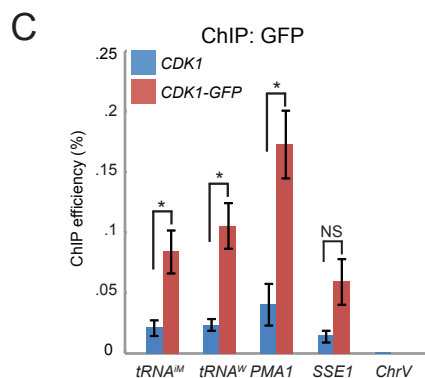

Supplement: Supplementary Data [file gky846_supplemental_files.zip › Suppl. Figure_S1.pdf]

Herrera\_Supplemental Figure S2

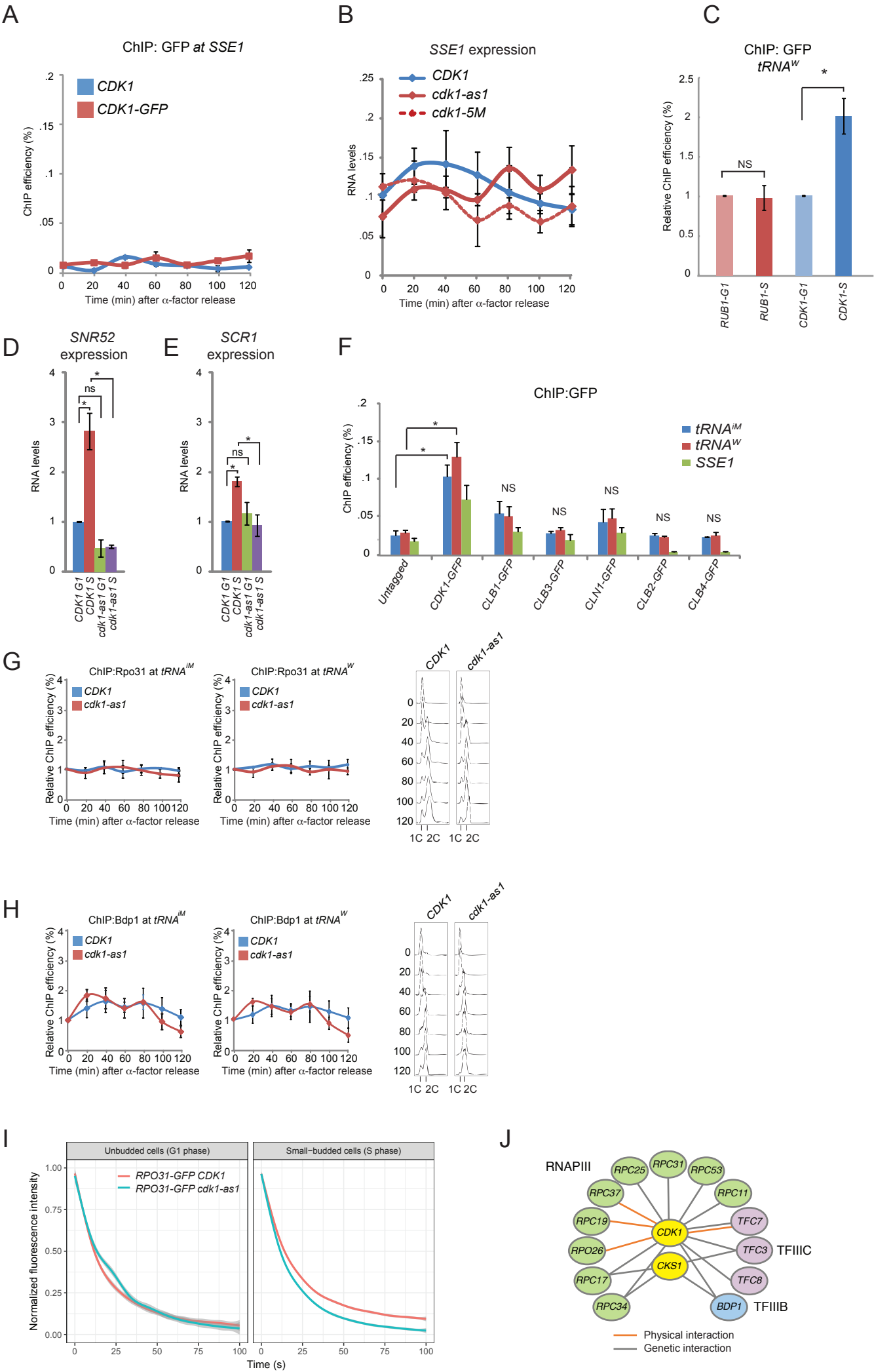

Supplement: Supplementary Data [file gky846_supplemental_files.zip › Suppl. Figure_S2.pdf]
